# The combined effect of physical activity and sedentary behavior on subclinical atherosclerosis: a cross-sectional study among Mexican Americans

**DOI:** 10.1186/s12889-019-6439-4

**Published:** 2019-02-06

**Authors:** Timothy J. Walker, Natalia I. Heredia, MinJae Lee, Susan T. Laing, Susan P. Fisher-Hoch, Joseph B. McCormick, Belinda M. Reininger

**Affiliations:** 1 Department of Health Promotion and Behavioral Sciences, Center for Health Promotion and Prevention Research, The University of Texas Health Science Center at Houston School of Public Health, 7000 Fannin St, Houston, TX 77030 USA; 2 Department of Health Disparities Research, The University of Texas MD Anderson Cancer Center, 1400 Pressler, Houston, TX 77030 USA; 3 Division of Clinical and Translational Sciences, Department of Internal Medicine, The University of Texas McGovern Medical School; Biostatistics/Epidemiology/Research Design (BERD) Core, Center for Clinical and Translational Sciences (CCTS), The University of Texas Health Science Center at Houston, 6410 Fannin St, Houston, TX 77030 USA; 4 Division of Cardiology, Department of Internal Medicine, McGovern Medical School at the University of Texas Health Science Center at Houston, 6431 Fannin St, Houston, TX 77030 USA; 5 Department of Epidemiology, Human Genetics and Environmental Sciences, The University of Texas Health Science Center at Houston School of Public Health, Brownsville Regional Campus, 1 W University Blvd, Brownsville, TX 78520 USA; 6 Department of Health Promotion and Behavioral Sciences, The University of Texas Health Science Center at Houston School of Public Health, Brownsville Regional Campus, 1 W University Blvd, Brownsville, TX 78520 USA

**Keywords:** Physical activity, Sedentary behavior, Atherosclerosis, Carotid intima-media thickness, Carotid plaque

## Abstract

**Background:**

Physical activity and sedentary behavior are considered independent risk factors for chronic disease. However, we do not fully understand their interrelation with key health outcomes such as subclinical atherosclerosis. This study examines the combined effects of sedentary behavior and physical activity on carotid intima-media thickness (cIMT) and presence of carotid plaque in a Mexican American population on the Texas-Mexico border.

**Methods:**

This cross-sectional study was conducted using retrospective data from a sample (*n* = 612) of participants from the Cameron County Hispanic Cohort. Carotid ultrasound was used to measure cIMT and presence of carotid plaque. Self-reported questionnaires were used to assess leisure time physical activity and sedentary behavior (TV/movie sitting and total sitting). A series of multivariable regression models were used to assess study aims. An interaction term between physical activity and sedentary behavior was included in models for each respective outcome. Models were controlled for demographic and health-related variables.

**Results:**

There were no significant associations found between physical activity, sedentary behavior and mean cIMT, or cIMT thickness ≥ 75th percentile for age and gender. However, there was a significant interaction between physical activity and TV/movie sitting with presence of carotid plaque. Participants who reported moderate levels of physical activity had significantly lower odds for presence of plaque compared to participants with no activity when TV/movie sitting time was ≤3 h per day. However, there was no significant difference in odds for presence of plaque between physical activity groups when TV/movie sitting exceeded 3 h/day. These results were consistent with models examining total sitting time.

**Conclusions:**

Our results indicate that for Mexican Americans, there is a combined effect of sedentary behavior and physical activity on presence of carotid plaque. Participating in moderate physical activity is optimal for having lower levels of carotid plaque in addition to avoiding excessive levels of TV/movie sitting (≥3 h/day) and/or total sitting (≥8.5 h/day).

## Background

Physical activity and sedentary behavior are considered distinct behaviors and independent risk factors for multiple health issues [1, 2]. Sedentary behavior is different from physical inactivity (insufficient levels of physical activity), where it has been defined as any waking behavior with an energy expenditure ≤1.5 metabolic equivalents while in a sitting or lying position [3]. Research examining the health impact of sedentary behavior has grown rapidly. However, there is insufficient evidence to determine the combined effects that various levels of physical activity and sedentary behavior may have on key health outcomes including cardiovascular disease [1]. Much of the existing research assessing the interrelation between physical activity and sedentary behavior has focused on all-cause mortality and cardiovascular disease mortality [4]. Results from these studies provide evidence to support that the relation between sedentary behavior and mortality varies by the amount of physical activity [4].

Given the long-term health effects of physical activity and sedentary behavior, it is important to examine its combined effects with earlier stages of cardiovascular disease for preventive strategies. Subclinical atherosclerosis can be measured by carotid ultrasonography and multiple studies have shown prediction of cardiovascular events and stroke by carotid intima-media thickness (cIMT) and/or presence of carotid plaque [5–10]. Presence of carotid plaque and cIMT are related, yet they capture different elements of cardiovascular health. As a result, these variables have been used independently or in an additive approach to predict cardiovascular risk [11].

Research examining the association between physical activity, sedentary behavior, and measures of subclinical atherosclerosis is less established where past studies in this area have reported mixed results [12–28]. These mixed findings could be a result of few studies assessing physical activity alongside sedentary behavior in adults [17, 19, 20] or studies equating sedentary behavior to inactivity [27]. A better understanding of the potential combined effects of physical activity and sedentary behavior on subclinical atherosclerosis will provide needed clarity about the potential negative effect of sitting across different levels of activity for a critical health outcome.

Furthermore, to our knowledge, no study has assessed both the association of physical activity and sedentary behavior with cIMT or carotid plaque within a Mexican American population. Hispanics generally have lower levels of physical activity than other racial/ethnic groups [29, 30]. Given that Hispanics are the largest minority group in the U.S. and Mexican Americans are the largest sub-group of Hispanics [31], it is important to determine whether modifiable behaviors such physical activity and sedentary behavior are associated with subclinical atherosclerosis in a Mexican American population. Therefore, the purpose of this study is to assess the combined effects of both sedentary behavior (sitting time) and physical activity on cIMT and carotid plaque in Mexican Americans on the Texas-Mexico border.

## Methods

### Study design and setting

A cross-sectional design was used to assess study aims. Data for analyses were from the Cameron County Hispanic Cohort (CCHC), a randomly selected cohort of Mexican-Americans living in Brownsville, Texas, which is a homogenous population located on the US/Mexico border [32]. Extensive clinical data and self-reported questionnaire data capturing demographic and health-related information were collected from CCHC participants. The Institutional Ethics Review Board of the University of Texas Health Science Center at Houston approved this study.

### Participants

Recruitment of cohort participants was completed by randomly selecting tract/blocks according to the 2000 Census as described in previous studies [32, 33]. Participants were 18 years or older and they were recruited from households where there could be multiple participants per household and from the same family. All participants gave written informed consent. Ongoing cohort data collection began in 2004, and a total of 4343 participants were considered for this analysis. In this study, we used de-identified data from a subset of CCHC participants who completed questionnaires and clinical studies relevant to research aims. Some clinical studies and questionnaires were introduced after the start of original data collection in 2004. Thus, data analyzed in this study were collected from March, 2010 to July, 2017.

### Measures

#### Carotid ultrasound evaluation

Starting in 2010, cohort participants were invited to complete a Carotid ultrasound at their first visit or when they came in for a follow-up visit. Carotid ultrasounds were conducted using the Siemens Acuson X300 ultrasound system (Malvern, PA) using a VF 13–5 linear array transducer. Guidelines from the American Society of Echocardiography consensus statement on subclinical vascular disease were used to inform the study protocol [34]. Details of imaging procedures have been described elsewhere [35]. Primary study outcomes included cIMT thickness and presence of carotid plaque. Two variables were used to represent cIMT thickness: a continuous variable of thickness measured in millimeters and a dichotomous variable of having a cIMT thickness ≥ 75th percentile for age and gender. Presence of carotid plaque was defined as having wall thickening in the carotid artery that was > 50% of the thickness of the surrounding wall [36]. The selected measures of cIMT and carotid plaque are subclinical markers of early atherosclerosis and valid predictors of future cardiovascular events [5–10]. A single blinded expert read all images and measurements with replicate readings performed on 5% of the cohort with results indicating an intraclass correlation value of 0.96.

#### Physical activity and sedentary behavior variables

The key independent variables were self-reported physical activity and time spent sitting watching TV/movies. Physical activity was measured using a modified version of the Godin Leisure-Time Exercise Questionnaire [37], the original version of which has been found to be a valid and reliable among Hispanics [38]. The physical activity questionnaire assessed participants’ intensity, frequency, and duration of leisure time physical activity. Participants were categorized into four groups based on the volume (time and intensity) of weekly activity using standard metabolic equivalent (MET) values, where 1 MET is equivalent to the rate of energy expenditure at rest. For example, moderate intensity activity corresponds with 3.0–5.9 METs. Thus, a moderate activity of 4 METs performed for 150 min per week equates to 600 MET minutes/week [39], which is consistent with the amount of activity recommended by the US Physical Activity Guidelines for Americans [40]. The grouping of the physical activity variable was informed by the US Physical Activity guidelines: no activity (0 MET min/wk), low activity (1–599 MET min/wk), moderate activity (600–1499 MET min/wk), and high activity (≥1500 MET min/wk). Based on scoring protocols, participants were excluded from analyses if they reported extreme physical activity values (≥7680 MET min/wk).

Time spent watching TV/movies was originally assessed in the cohort survey using a single question asking participants to report the frequency and duration of sitting for TV or movies during the last 7 days. To improve the assessment of sedentary behavior, the multi-context sitting time questionnaire (MSTQ) was later added to the cohort survey as a more comprehensive measure with acceptable validity and reliability [41]. The MSTQ captures total sitting time in a typical week- and weekend day across the following domains: occupational, leisure-time, and transportation. Within the leisure-time domain, there is a question specific to sitting time while watching TV or movies. Therefore, sitting time for TV/movies was assessed by harmonizing the original survey question and the MSTQ question. More specifically, week- and weekend day TV/movie sitting (assessed by the MSTQ) were combined using a weighted average to capture total minutes per week of TV/movie sitting. The variable was then merged with the original TV/movie sitting question and rescaled to capture hours per day of TV/movie sitting to facilitate the interpretation of findings. A total sitting time (hours/day) variable was also created for the subsample of cohort participants who completed the MSTQ for a secondary analysis. The MSTQ includes a question about whether answers reflect a typical week of sitting where participants who responded “no” were excluded from analyses. Participants were also excluded if they reported daily sitting values > 24 h.

#### Covariates

To control for confounding, variables from self-report questionnaires and laboratory studies were considered for inclusion in analyses based on their theoretical importance and previous research [35]. Demographic variables included gender, age, employment status (employed/unemployed), marital status (single/married, divorced/separated), and years of education. Smoking status was assessed by asking participants whether they smoke cigarettes. Alcohol was assessed by asking participants the number of drinks consumed per week (12 oz. beer, 8 oz. wine cooler, 4 oz. wine, or 1 oz. liquor). Drinks per week were converted to drinks per day where heavy alcohol use was considered to be more than two drinks per day for males and more than one drink per day for females [42]. Dietary patterns were assessed using the Healthy/Unhealthy Eating Indices where participants self-reported the number of times they ate a healthy (or unhealthy) food the previous day [43]. Healthy foods included fruit, fruit juice, baked/grilled poultry, baked/grilled fish, whole grain breads, whole grain cereals, eggs, orange vegetables, salad, beans, and other vegetables where an index score was created from responses with a possible range from 0 to 55. Unhealthy foods included regular sodas, sweetened drinks/sports drinks, sweetened tea, frozen desserts, pastries, non-chocolate candy, white bread, red and processed meats, fried meat, and French fries/chips where the index score could range from 0 to 50. Having a history of stroke, heart attack, and angina (yes/no) were also assessed by a series of self-reported questions (e.g. have you ever been diagnosed or told by a doctor you had a stroke?).

Participants also completed a series of anthropometric measures and laboratory studies. Height and weight were measured to calculate body mass index (BMI). Systolic and diastolic blood pressure were measured by taking the average of the second and third readings occurring 5 minutes apart. Cohort participants provided blood samples for the following laboratory studies: fasting lipid panel (to assess cholesterol and triglycerides), glycated hemoglobin (HbA1c), fasting plasma glucose, and fasting serum insulin. To assess insulin resistance, the homeostasis model assessment insulin resistance index (HOMA-IR) was calculated using fasting glucose and insulin [44].

### Statistical analyses

Descriptive statistics were assessed across the two dichotomous outcome variables. A multivariable linear regression model was used to determine whether physical activity and TV/movie sitting were significantly associated with mean cIMT. Two multivariable logistic regression models were used to determine whether physical activity and TV/movie sitting were significantly associated with 1) presence of plaque, and, 2) cIMT thickness ≥ 75th percentile. A first set of models only included the physical activity and sedentary behavior as independent variables. A next set of models adjusted for age and gender, and third set adjusted for age, gender, and additional demographic and health variables. The following demographic and health-related variables were considered for inclusion in models if they were statistically significant: employment status, marital status, years of education, healthy/unhealthy food indices, smoking status, heavy alcohol use, history of stroke, history of heart attack, history of angina, BMI, total cholesterol, triglycerides, systolic and diastolic blood pressure, HbA1c, and HOMA_IR. Models were also adjusted for having multiple participants from the same household by using generalized estimating equations (GEE) to control for clustering. An interaction term between physical activity and sedentary behavior was included in each model to assess whether the relation between sedentary behavior and outcomes were different across physical activity levels. A secondary analysis was also performed following the procedures previously described using the total sitting time variable with the subsample of participants who completed the MSTQ. All analyses were conducted using Stata 13.0.

## Results

**Fig. 1:**
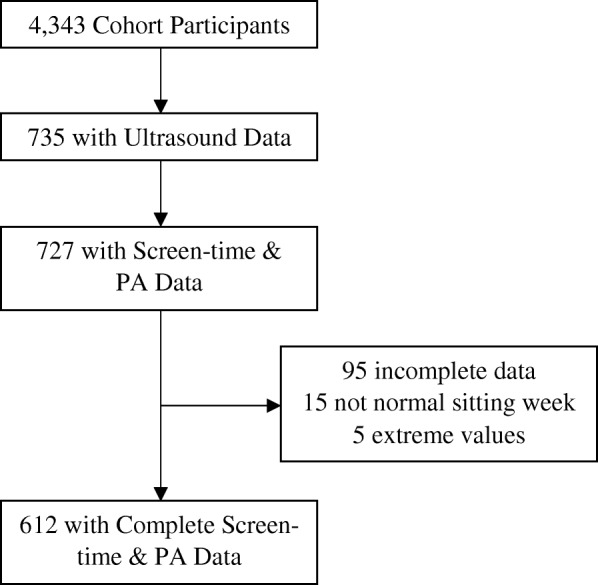
Analytic Sample

There were 727 cohort participants who completed a carotid ultrasound and self-report surveys that included the TV/movie sitting questions. However, 95 participants were missing sedentary behavior data and 22 were excluded from analyses due to extreme values or reporting a non-normal week of sitting (Fig. 1). Thus, there were 612 participants with complete data for all three dependent and key independent variables from 459 unique households (Table 1). Some participants were missing data for other demographic and health-related control variables (Table 1) leading to sample sizes ranging from 592 to 607 in fully adjusted models. The majority of the sample was female (67.6%) and middle-aged at the time of the ultrasound (mean age 50.4 ± 14.1 years; Table 1). Most participants were not doing any leisure-time physical activity (64.4% reported no physical activity) while the average time spent sitting for TV/movies was about 1.8 h per day. About 16% of participants had cIMT ≥75% while about 24% had presence of carotid plaque. There was a high prevalence of obesity (51.4%) and other cardiovascular disease risk factors in the sample (32.4% were hypertensive and 19.4% had diabetes). These health trends were similar when compared to the total cohort of participants (*n* = 4343): obesity, 50.9%; hypertension, 31.2%; and diabetes, 20.2%. However, in the complete cohort, there was a slightly lower percentage of females (64.0%) and mean age (45.3 ± 17.2 years).

**Table 1:** Descriptive statistics for total sample and across cIMT ≥75% and presence of carotid plaque variables

Variable, M (SD)	Total *N* = 612	cIMT ≥75%		Presence of Carotid Plaque	
		Normal *N* = 512	Abnormal *N* = 100	No Plaque *N* = 462	Plaque *N* = 150
Physical Activity and Sedentary Behavior Variables					
Physical Activity (n, %)					
No Activity	394 (64.4)	324 (63.3)	70 (70.0)	277 (59.9)	117 (78.0)
Low Activity	49 (8.0)	44 (8.6)	5 (5.0)	39 (8.4)	10 (6.7)
Moderate Activity	81 (13.2)	71 (13.9)	10 (10.0)	71 (15.4)	10 (6.7)
High Activity	88 (14.4)	73 (14.3)	15 (15.0)	75 (16.2)	13 (8.7)
TV/Movie Sitting Time (hr./day)	1.8 (1.6)	1.7 (1.6)	2.0 (1.7)	1.7 (1.5)	2.1 (1.8)
Demographic Variables					
Female (n, %)	414 (67.6)	363 (70.9)	51 (51.0)	329 (71.2)	85 (56.7)
Age at cIMT	50.4 (14.1)	49.5 (14.5)	54.9 (10.6)	46.9 (13.5)	61.0 (9.9)
Employed (n, %)	309 (50.5)	259 (50.6)	50 (50.0)	254 (55.0)	55 (36.7)
Marital Status (n, %)					
Single	98 (16.0)	91 (17.8)	7 (7.0)	86 (18.6)	12 (8.0)
Married	406 (66.3)	333 (65.0)	73 (73.0)	304 (65.8)	102(68.0)
Divorced/Separated	108 (17.7)	88 (17.2)	20 (20.0)	72 (15.6)	36 (24.0)
Years Education	11.1 (5.5)	11.3 (5.6)	10.1 (4.7)	11.5 (5.4)	9.8 (5.5)
Health Related Variables					
Smoking (n, %)					
Never	202 (33.0)	161 (31.4)	41 (41.0)	150 (32.5)	52 (34.6)
Sometimes	135 (22.1)	110 (21.5)	25 (25.0)	93 (20.1)	42 (28.0)
Other	275 (44.9)	241 (47.1)	34 (34.0)	219 (47.4)	56 (37.3)
Heavy Alcohol Use (n, %)	20 (3.3)	11 (2.2)	9 (9.0)	14 (3.0)	6 (4.0)
BMI (kg/m2)	30.9 (6.3)	30.8 (6.4)	31.1 (5.4)	30.9 (6.4)	30.6 (5.7)
Total Cholesterol (mg/dL)	182.4 (37.9)	181.1 (36.4)	188.9 (44.5)	181.0 (35.2)	186.8 (45.0)
Triglycerides (mg/dL)	146.7 (89.5)	142.5 (88.9)	168.4 (89.9)	142.5 (90.4)	159.9 (85.6)
HDL-Cholesterol (mg/dL)	45.9 (13.2)	46.3 (13.0)	43.9 (13.6)	45.9 (13.0)	45.9 (13.8)
LDL-Cholesterol (mg/dL)	108.0 (31.8)	107.2 (30.7)	112.1 (36.8)	107.2 (30.3)	110.5 (36.0)
Systolic Blood Pressure (mmHg)	120.1 (18.0)	118.1 (16.9)	130.6 (20.1)	117.2 (15.8)	129.1 (21.1)
Diastolic Blood Pressure (mmHg)	72.4 (9.3)	71.9 (8.8)	74.9 (11.0)	72.0 (8.7)	73.7 (10.8)
Insulin (mg/dL)	13.1 (9.7)	13.0 (9.7)	13.6 (9.9)	13.0 (9.4)	13.4 (10.5)
Mean Glucose (mg/dL)	116.9 (38.0)	112.6 (33.4)	138.1 (50.6)	111.5 (31.9)	133.5 (49.1)
HOMA_IR	3.5 (2.9)	3.4 (2.8)	4.0 (3.4)	3.3 (2.6)	3.9 (3.7)
Hba1C (%)	5.8 (1.3)	5.7 (1.22)	6.4 (1.5)	5.7 (1.2)	6.3 (1.5)
Healthy Food Index	7.2 (3.6)	7.2 (3.5)	7.4 (3.8)	7.1 (3.6)	7.4 (3.5)
Unhealthy Food Index	4.7 (3.2)	4.7 (3.2)	4.8 (3.2)	4.8 (3.3)	4.4 (2.7)
History of Stroke (n, %)	15 (2.4)	11 (2.1)	4 (4.0)	9 (1.9)	6 (4.0)
History of Heart Attack (n, %)	9 (1.5)	6 (1.2)	3 (3.0)	4 (0.9)	5 (3.3)
History of Angina (n, %)	10 (1.6)	8 (1.6)	2 (2.0)	5 (1.1)	5 (3.3)

Cholesterol through Unhealthy Food Index had sample sizes ranging from 419 to 612

### cIMT model results

Results from the unadjusted linear model revealed a statistically significant inverse association between moderate and high levels of physical activity and mean cIMT (Table 2). However, after adjusting for age and gender, the associations between physical activity levels and mean cIMT decreased in value and were no longer statistically significant. There was no significant association between TV/movie sitting time and mean cIMT in the unadjusted or adjusted models (Table 2). Further, there was no evidence of an interaction between physical activity and TV/movie sitting time in relation to mean cIMT (results not shown). Results from models using the dichotomous cIMT ≥75% variable were relatively consistent with results from the linear cIMT models where there were no significant associations in unadjusted or adjusted models (Table 2), nor was there a significant interaction between physical activity and sedentary behavior (results not show).

**Table 2:** Unadjusted and adjusted multivariable models with 4-category physical activity variable and TV/movie related sitting time

	Mean difference of cIMT (95% CI)	cIMT ≥75% (OR, 95% CI)	Presence of Plaque (OR, 95% CI)
Physical Activity (referent = no activity)^a^			
Low Activity	−0.04 (− 0.08–0.001)	0.51 (0.18–1.47)	0.64 (0.31–1.31)
Moderate Activity	− 0.05 (− 0.08--0.01)*	0.64 (0.31–1.30)	0.33 (0.17–0.63)*
High Activity	− 0.06 (− 0.09--0.03)*	0.96 (0.53–1.73)	0.42 (0.22–0.77)*
TV/Movie Sitting Time (hrs/day)	0.001 (− 0.01–0.01)	1.06 (0.93–1.20)	1.14 (1.01–1.27)*
Physical Activity (referent = no activity)^b^			
Low Activity	−0.02 (− 0.04–0.01)	0.53 (0.18–1.54)	0.74 (0.34–1.62)
Moderate Activity	− 0.02 (− 0.05–0.01)	0.69 (0.34–1.40)	0.35 (0.16–0.74)*
High Activity	− 0.01 (− 0.04–0.02)	1.10 (0.58–2.08)	0.60 (0.30–1.22)
TV/Movie Sitting Time (hrs/day)	−0.002 (− 0.01–0.01)	1.02 (0.90–1.16)	1.10 (0.97–1.25)
Physical Activity (referent = no activity)^c^			
Low Activity	−0.002 (− 0.03–0.02)	0.51 (0.13–1.95)	0.94 (0.42–2.08)
Moderate Activity	−0.009 (− 0.04–0.02)	0.87 (0.42–1.81)	0.35 (0.16–0.76)*
High Activity	− 0.009 (−0.04–0.02)	1.40 (0.73–2.68)	0.60 (0.28–1.26)
TV/Movie Sitting Time (hrs/day)	−0.002 (− 0.01–0.005)	0.97 (0.85–1.14)	1.10 (0.96–1.25)

a, unadjusted model (n = 612); b, adjusted for age and gender (*n* = 612); c, fully adjusted model

Fully adjusted mean cIMT model included age, gender, cholesterol, systolic blood pressure, diastolic blood pressure and HBA1c (*n* = 593)

Fully adjusted cIMT ≥75% model included age, gender, cholesterol, systolic blood pressure, heavy alcohol use, and HBA1c (*n* = 592)

Fully adjusted plaque model included age, gender, cholesterol, and systolic blood pressure (n = 607)

hrs/day is the weighted average of sitting on a typical weekday and weekend day

*, *p* < 0.05

### Carotid plaque model results

Results from the unadjusted model revealed a statistically significant inverse associations between moderate and high physical activity levels and presence of carotid plaque, and, a statistically significant direct association between TV/movie sitting time and presence of carotid plaque (Table 2). Results from the age and gender adjusted, and fully adjusted models were mostly consistent with the unadjusted model where there was a statistically significant inverse relation between moderate physical activity and presence of carotid plaque (Table 2). However, adjusted model results revealed non-significant trends for an inverse relation between high physical activity and presence of carotid plaque, and, a direct relation between TV/movie sitting time and presence of carotid plaque (Table 2).

When testing the interaction between physical activity and TV/movie sitting, model results revealed a significant interaction suggesting the relation between physical activity and carotid plaque differed across levels of TV/movie sitting (Table 3). Participants who reported no physical activity had consistent odds for presence of plaque across all levels of TV/movie sitting. However, participants who reported moderate physical activity had lower odds for plaque across low levels of TV/movie sitting, but as TV/movie sitting time reached higher levels (≥3 h/day) the odds for plaque increased. When comparing odds between activity levels, participants who reported moderate levels of physical activity had significantly lower odds for presence of plaque compared to participants with no activity when TV/movie sitting time was ≤3 h per day (Table 3). However, when TV/movie sitting time exceeded 3 h/day, there was no significant difference in odds for presence of plaque between those who reported moderate activity versus those who reported no activity. Notably, for those who reported high activity levels, adjusted odds were relatively stable across all TV/movie sitting times with no statistically significant differences when compared to those with no activity.

**Table 3:** Physical activity effect on carotid plaque across levels of TV/movie sitting time (*n* = 607)

Physical activity x Sitting Time	Adjusted OR (95% CI)	*p*-value
Low Activity vs No Activity		
Sedentary at 0.33 h/day	0.73 (0.28–1.86)	0.507
Sedentary at 1.06 h/day	0.83 (0.37–1.87)	0.662
Sedentary at 1.96 h/day	0.99 (0.43–2.27)	0.979
Sedentary at 4.01 h/day	1.46 (0.34–6.14)	0.610
Moderate Activity vs No activity		
Sedentary at 0.33 h/day	0.19 (0.07–0.55)	0.002
Sedentary at 1.06 h/day	0.24 (0.09–0.62)	0.003
Sedentary at 1.96 h/day	0.32 (0.13–0.75)	0.009
Sedentary at 4.01 h/day	0.58 (0.23–1.43)	0.237
High Activity vs No activity		
Sedentary at 0.33 h/day	0.65 (0.22–1.85)	0.416
Sedentary at 1.06 h/day	0.62 (0.26–1.47)	0.279
Sedentary at 1.96 h/day	0.59 (0.29–1.23)	0.163
Sedentary at 4.01 h/day	0.54 (0.19–1.53)	0.245

Adjusted for age, gender, cholesterol, and systolic blood pressure

### Secondary analysis

Using total sitting time from the subsample of participants who completed the MSTQ led to a smaller analytic sample ranging from 336 to 340 participants in fully adjusted models. Results from these fully adjusted models were also similar to results from the primary analysis. There were no significant associations between physical activity and total sitting time with mean cIMT or cIMT ≥75% (Table 4). When assessing relations with plaque, there was a non-significant association between total sitting time and presence of carotid plaque while there was a significant inverse association between moderate physical activity and presence of carotid plaque (Table 4).

**Table 4:** Adjusted multivariable GEE models with total sitting time (hrs/day)

	Adjusted Mean difference of cIMT (95% CI)	cIMT ≥75% (adjusted OR, 95% CI)	Presence of Plaque (adjusted OR, 95% CI)
Physical Activity (referent = no activity)			
Low Activity	−0.009 (− 0.04–0.02)	0.54 (0.09–3.41)	1.18 (0.35–3.94)
Moderate Activity	0.010 (− 0.03–0.05)	1.17 (0.45–3.07)	0.27 (0.07–0.99)*
High Activity	− 0.014 (− 0.05–0.02)	1.28 (0.49–3.39)	0.41 (0.14–1.15)
Total Sitting Time (hrs/day)	0.001 (−0.003–0.004)	0.95 (0.89–1.02)	1.02 (0.94–1.09)

cIMT model adjusted for age, gender, systolic blood pressure, diastolic blood pressure and HBA1c (*n* = 336)

cIMT ≥75% model adjusted for age, gender, cholesterol, systolic blood pressure, and HBA1c (n = 336)

Plaque model adjusted for age, gender, and systolic blood pressure (*n* = 340)

hrs/day is the weighted average of sitting on a typical weekday and weekend day

*, *p* < 0.05

When testing the interaction between physical activity and total sitting time with the presence of plaque outcome, results were relatively consistent with the TV/movie sitting time model (Table 5). Notably, participants who reported moderate physical activity had significantly lower odds for carotid plaque across lower levels of total sitting compared to participants who reported no activity (Table 5). However, at high levels of total sitting time (≥8.5 h/day), there was no significant difference between adjusted odds of carotid plaque between participants who reported moderate activity to participants who reported no activity. Similar to the TV/movie sitting time model, there was no statistically significant difference in the adjusted odds for carotid plaque between participants who reported high activity versus participants who reported no activity across all levels of sitting time. However, there was a more prominent inverse trend for participants in the high activity group where a greater amount of total sitting was associated with lower adjusted odds for carotid plaque.

**Table 5:** Physical activity effect on carotid plaque across levels of total sitting time (n = 340)

Physical Activity x Sitting Time	Adjusted OR (95% CI)	p-value
Low Activity vs No Activity		
Sedentary at 1.99 h/day	1.31 (0.24–7.19)	0.754
Sedentary at 4.35 h/day	1.25 (0.32–4.82)	0.750
Sedentary at 7.68 h/day	1.16 (0.33–4.02)	0.818
Sedentary at 14.64 h/day	0.99 (0.09–10.52)	0.995
Moderate Activity vs No activity		
Sedentary at 1.99 h/day	0.03 (0.002–0.35)	0.005
Sedentary at 4.35 h/day	0.06 (0.01–0.46)	0.007
Sedentary at 7.68 h/day	0.18 (0.36–0.85)	0.030
Sedentary at 14.64 h/day	1.56 (0.21–11.75)	0.667
High Activity vs No activity		
Sedentary at 1.99 h/day	1.50 (0.27–8.49)	0.645
Sedentary at 4.35 h/day	0.94 (0.29–3.05)	0.923
Sedentary at 7.68 h/day	0.49 (0.18–1.31)	0.155
Sedentary at 14.64 h/day	0.12 (0.01–1.83)	0.129

Adjusted for age, gender, and systolic blood pressure

## Discussion

This study examined the relations between physical activity, sedentary behavior, and subclinical atherosclerosis among a cohort of Mexican Americans. Results provide evidence that participating in moderate levels of physical activity are associated with lower odds for carotid plaque across low to moderate levels of sitting. However, at high levels of sitting, there appears to be no difference in odds for carotid plaque between physical activity levels. These results suggest participating in moderate physical activity and avoiding excessive levels of sitting time are associated with the lowest odds for carotid plaque compared to other combinations of physical activity and sedentary behavior. There was no evidence that physical activity or sedentary behavior were associated with mean cIMT (after adjusting for age and gender) among Mexican Americans.

Results were relatively consistent between the primary and secondary analyses. However, for those in the high physical activity group, a more prominent inverse trend between total sitting and adjusted odds for plaque appeared to be present in the secondary analysis. Because the secondary analysis was limited to those who completed the MSTQ, the study sample was reduced to 340 participants. This led to a low number of participants who had carotid plaque who were also moderately (*n* = 3) or highly active (*n* = 6). This likely contributed to a more exaggerated inverse association in the highly active group than what was observed in the primary analysis. In addition, it may be why there was a more exaggerated increase in the odds of plaque for the moderate activity group at high values of sitting. Among participants who had complete sedentary behavior data, TV/movie sitting was moderately correlated with total sitting time (r = 0.40) and on average made up 35% of total sitting time. Thus, TV/movie sitting is one of the more prominent sitting domains for this sample of Mexican Americans and likely served as an adequate proxy for total sitting time in this group.

Previous studies assessing the relation between physical activity and measures of subclinical atherosclerosis (cIMT and/or plaque) have reported inconsistent findings. Some studies have reported a significant inverse association between physical activity and cIMT [14, 15, 25, 27, 45] while others have reported no association [13, 16, 21, 23]. Results from a literature review [45] suggested inconsistent findings were likely due to a wide variability between study populations and weak measures of physical activity with the use of arbitrary cut-offs.

The lack of association between physical activity and cIMT in our study may be due to multiple factors. First, physical activity and sedentary behavior were measured at one time point capturing a participant’s typical week of activity. This may not be representative of one’s physical activity profile during adolescence or other periods of life, which may be an important factor in slowing the progression of atherosclerosis [23, 46, 47]. Second, the association between physical activity and mean cIMT was no longer significant after controlling for age and gender. Thus, cIMT may be driven by other risk factors and not physical activity and sedentary behavior levels [5, 7]. Third, there was a relatively small number of people in respective physical activity groups who had a cIMT ≥75%, which likely also contributed to the non-significant findings.

Our results did indicate a relation between physical activity and plaque even after controlling for other confounders. The association with plaque but not cIMT may be an indication that physical activity is associated with a more advanced stage of the atherosclerotic process. It is also possible that different risk factors are associated with cIMT and plaque suggesting they are related but not identical components in the development of atherosclerosis [5, 7, 48]. Studies indicate carotid plaque is related to atherosclerotic processes, which include inflammation, endothelial dysfunction, and oxidative stress whereas cIMT represents thickening of the smooth muscle and is primarily influenced by age and hypertension [7, 11, 49]. Thus, physical activity and sedentary behavior may be associated with plaque (and not cIMT) due to their influence on these atherosclerotic processes [50–52].

Notably, only those who participated in moderate (and not high) levels of physical activity had lower odds for plaque indicating physical activity and presence of plaque may not be linearly related. Other studies have reported a non-linear relation between physical activity and measures of atherosclerosis where it was suggested that very high levels of physical activity were associated with low-grade inflammation, which could exert an atherogenic effect [23, 53]. It is also possible the non-linear trend we observed could be due to the relatively small sample size or limitations in using a self-report measure of physical activity, where people are known to over report activity levels [54], which may lead to some misclassifications in the physical activity categories.

Few studies have examined sedentary behavior as a risk factor for subclinical atherosclerosis where some studies have reported a direct association [14, 17] while others have reported no association [19, 20]. Sedentary behavior has been inconsistently operationalized throughout the literature (e.g., objectively measured, self-report, TV-sitting, inactivity, etc.) which is likely contributing to the inconsistent findings. To our knowledge, no studies have examined the interaction between physical activity and sedentary behavior to gain a better understanding of how the combined effect of these variables may be associated with measures of subclinical atherosclerosis. As previously reported, our results indicate moderate activity is associated with favorable outcomes. Our findings expand on the current literature by suggesting sitting time is not an additional risk factor for cIMT for people who are participating in no or low levels of physical activity. However, for those who participate in moderate physical activity, excessive levels of sitting time appear to contribute to higher odds for carotid plaque.

There are study limitations that need to be considered when interpreting results. First, this was a cross-sectional study that used self-report measures for physical activity and sedentary behavior. Even though the physical activity measure has been validated in a similar study population, we used a modified measure, which lacks additional validity testing. In addition, the sedentary behavior measure was changed during the study and later combined into single measures. Thus, we harmonized two different measures to capture TV/movie sitting time to maximize the study sample for the primary analysis. Further, the primary analysis was limited to TV/movie sitting, which serves as a proxy measure for total sitting levels. There are also some additional confounding factors we did not control for. For example, we did not take into account the potential impact of genetic risk on atherosclerosis and thus future studies should consider this factor. We also tried to control for diet using general variables to assess healthy and unhealthy eating, although results indicated the diet variables were not significantly contributing to models. A diet variable more focused on healthy eating to support cardiovascular health could have been more meaningful. Last, the majority of the sample was not physically active. Thus, there are a relatively small number of participants in the physical activity categories, which may impact the findings.

This study does have several strengths. First, it assessed relations among a homogeneous population of Hispanics. To our knowledge, this is the first study to examine physical activity and sedentary behavior with subclinical atherosclerosis among this population. In addition, our study focused on the interplay between physical activity and sedentary behavior to gain a better understanding about the activity profile rather than isolated, independent effects of sitting and physical activity. We also conducted a secondary analysis to assess total sitting time among a subsample of participants with a comprehensive measure of sedentary behavior. Results from the primary and secondary analyses were consistent providing additional support for findings. Lastly, even though we did not control for genetic risk and a specific diet to support cardiovascular health, analyses controlled for many potential confounders related to demographic, behavioral, and other health-related variables.

## Conclusions

Promoting moderate physical activity and avoiding sedentary behavior needs to remain a public health priority given the numerous health benefits including its association with subclinical atherosclerosis. This is especially true among ethnic sub-groups, such as Mexican Americans, which is the largest sub-group of Hispanics in the US who often experience health disparities. Our results support that participating in moderate physical activity is optimal for having lower levels of carotid plaque in addition to avoiding excessive levels of total sitting (≥8.5 h/day) and/or TV/movie sitting (≥3 h/day). These findings are largely consistent with recommendations from the 2018 US Physical Activity Guidelines for Americans where adults are recommended to participate in at least 150 min of moderate aerobic activity per week and reduce levels of sitting [40]. However, we provide further evidence that when participating in moderate activity, health risks associated with sedentary behavior occur at very high levels of sitting per day. Thus, interventions focused on cardiovascular health can better help people achieve the associated health benefits by promoting a more focused message of 150 min of moderate aerobic activity per week and avoiding TV sitting for more than 3 h/day or total sitting for more than 8.5 h/day. Future research should continue to examine how the physical activity profile (both activity levels and sitting time) can impact other health outcomes such as coronary heart disease events and how genetics may play a role in these relationships.

## Abbreviations

BMI: Body mass index
CCHC: Cameron County Hispanic Cohort
cIMT: Carotid intima-media thickness
GEE: Generalized estimating equations
HOMA-IR: Homeostasis model assessment insulin resistance index
MET: Metabolic equivalent
MSTQ: Multi-context sitting time questionnaire
